# Customized Connective Tissue Graft Technique for Optimizing Interproximal Attachment Gain in Regenerative Treatment of Infrabony Defects: A Case Report With 36‐ to 56‐Month Follow‐Up

**DOI:** 10.1111/jerd.70061

**Published:** 2025-11-26

**Authors:** Michel Bravard, Kevimy Agossa, Hom‐Lay Wang

**Affiliations:** ^1^ Private Practice Lyon France; ^2^ Department of Periodontics and Oral Medicine University of Michigan School of Dentistry Ann Arbor Michigan USA; ^3^ University and CHU of Lille, School of Dentistry Department of Periodontology Lille France

**Keywords:** case series, connective tissue transplantation, gingival recession, guided tissue regeneration, periodontal attachment loss, periodontal pocket surgery, periodontitis

## Abstract

**Objective:**

The aim of this retrospective case report was to evaluate the long‐term effect of a customized connective tissue graft technique (c‐CTG) aimed at preserving or enhancing buccal and interproximal soft tissues in the context of regenerative treatment of infrabony defects in the esthetic zone.

**Clinical Considerations:**

Three patients presenting with a single, deep periodontal infrabony defect between the maxillary central incisors were treated using the c‐CTG. All sites demonstrated regenerative success, defined as a clinical attachment gain of ≥ 3 mm and probing depth ≤ 4 mm, maintained over a 3‐ to 5‐year follow‐up period. In Case 1, complete resolution of pre‐existing buccal recession was observed, along with a 2 mm increase in papilla height. Case 2 showed partial closure of a postoperative black triangle, with progressive papilla gain between 6 and 24 months. In Case 3, the initial interproximal gingival crater evolved into a positive soft tissue architecture that was maintained over the long term.

**Conclusion:**

Based on the findings, the c‐CTG appears to be a feasible approach for achieving both predictable regenerative outcomes and long‐term enhancements in both buccal and interdental soft tissue contours.

## 1 Introduction

Periodontal regenerative therapies (PRTs) are effective in improving clinical attachment level (CAL), reducing probing pocket depth (PPD), and thus enhancing long‐term prognosis of teeth presenting with infrabony defects [1, 2, 3]. However, postoperative soft tissue remodeling frequently results in gingival recession (GR), particularly in sites with a thin gingival phenotype or non‐supportive defect anatomy [4, 5]. Interproximal GR can lead to open embrasures or “black triangles,” which compromise smile esthetics, reduce patient satisfaction, and may limit the perceived success of PRT [6, 7].

Efforts to mitigate GR have included the use of papilla preservation flaps, which aim to minimize trauma to interproximal tissues and have demonstrated improved soft tissue stability compared to conventional access flaps [8]. The adjunctive use of bone grafts as a scaffold has also been associated with reduced interproximal tissue collapse [4, 5, 9]. Despite these advances, a recent systematic review reported a clinically significant increase in GR (≥ 1 mm) in 35 of 88 studies on PRT for infrabony defects, highlighting the persistent challenge of achieving optimal soft tissue outcomes [3].

The soft tissue wall technique, which involves the placement of a connective tissue graft (CTG) to compensate for buccal bone deficiencies, has shown potential for enhancing buccal soft tissue contours [10, 11, 12, 13]. However, its impact on interproximal tissues appears negligible—likely due to inadequate structural support for the interdental papilla. For instance, Trombelli et al. reported no significant difference in interproximal GR at 6 months, between sites treated with or without CTG, using a single flap approach combined with bone graft and biologics [14]. Similarly, our own one‐year case series using a comparable PRT protocol demonstrated no improvement in interproximal soft tissue levels, despite buccal enhancement [15].

To address this clinical limitation, we present a customized connective tissue graft (c‐CTG) technique in which the CTG is contoured and extended into the interproximal space to provide mechanical support at the base of the papilla. This modification aims to enhance the stability and preservation of both buccal and interproximal soft tissues, potentially improving esthetic outcomes. The present retrospective case report aims to illustrate the proposed approach and present clinical and radiographic evidence of successful outcomes in the esthetic zone, with follow‐up extending over a 3‐ to 5‐year period.

## 2 Materials and Methods

This case study adheres to the Case Report (CARE) guidelines [16].

### 2.1 Clinical Presentation

Three non‐smoking female patients (aged 47, 50, and 76 years at the time of surgery) were treated in a private periodontal practice, between June 2020 and March 2022. Each patient presented with a single, deep periodontal infrabony defect (≥ 3 mm depth on radiographic evaluation) localized between the maxillary central incisors.

**FIGURE 1:**
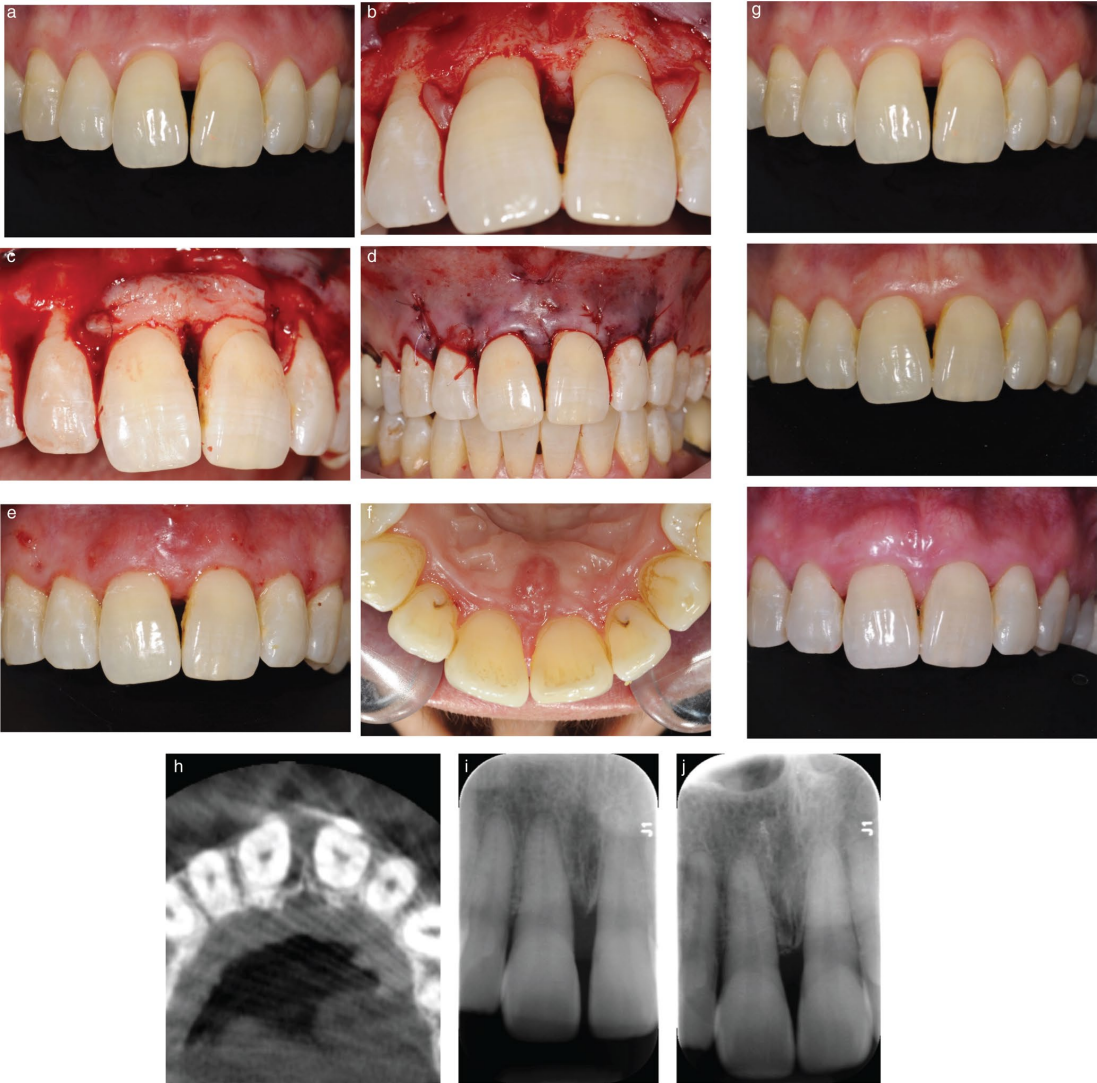
Case 1 (a) Preoperative view showing loss of the papilla, buccal recession, and a diastema. Infrabony defect following granulation tissue removal (b), placement of a contoured CTG (c), and flap closure (d). (e, f) Postoperative views at 15 days on the buccal and palatal aspects, showing maintained primary closure. (f) Soft tissue outcomes from baseline to 12 and 56 months, showing complete coverage of the buccal recession, an increase in papilla height, and spontaneous closure of the diastema. (g–i) Radiographic follow‐up (baseline CBCT and periapical radiographs at baseline and 56 months) showing complete bone fill.

**FIGURE 2:**
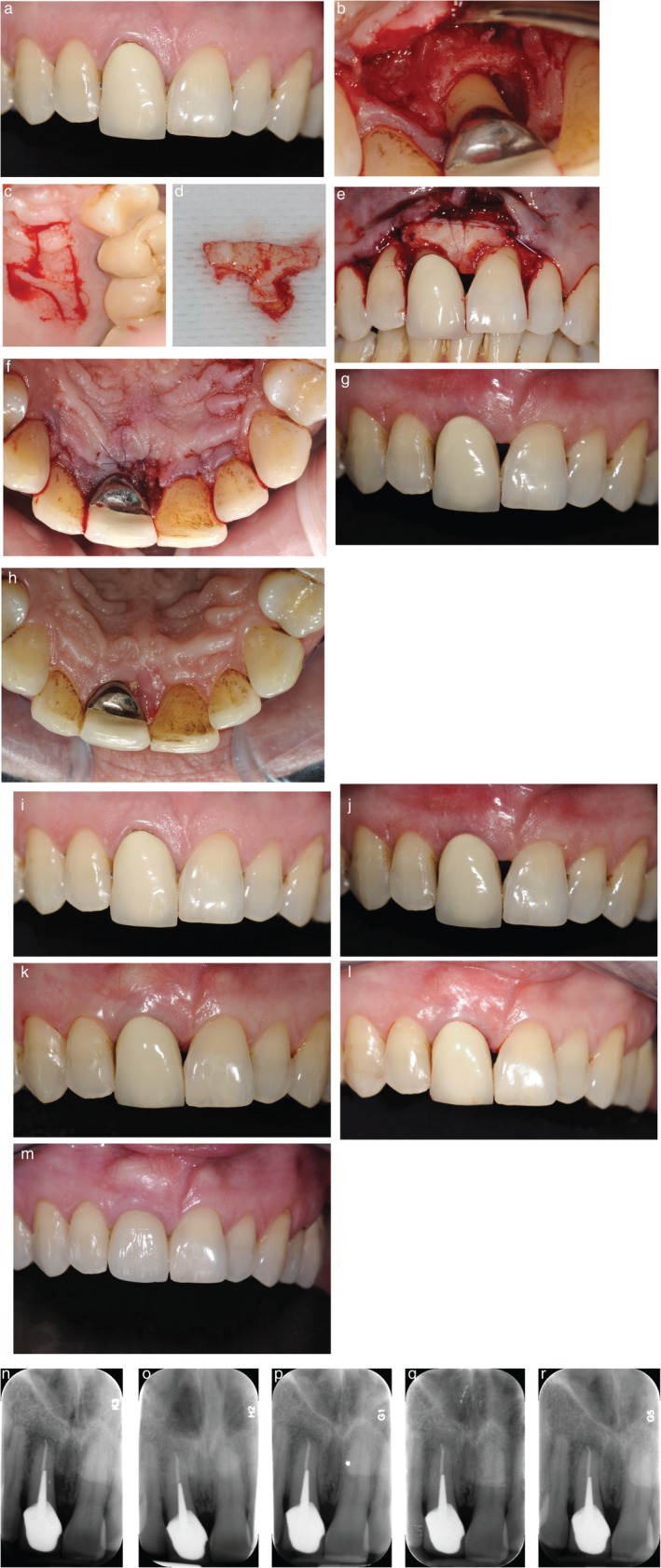
Case 2 (a) Preoperative view showing prosthetic crown malposition and minor papillary deficiency. Infrabony defect after degranulation (b); harvesting of a T‐shaped palatal graft (c, d); CTG placement with additional compressive suture to enhance graft stability (e). (f) Immediate postoperative view showing primary closure. (g, h) Postoperative views at 15 days on the buccal and palatal aspects, showing maintained closure and residual black triangle. (i–m) Soft tissue maturation from baseline to 6, 12, and 55 months; the prosthetic crown was replaced at 24 months. (n–r) Radiographic follow‐up from baseline to 6, 12, 24, and 55 months showing bone fill and long‐term stability.

**FIGURE 3:**
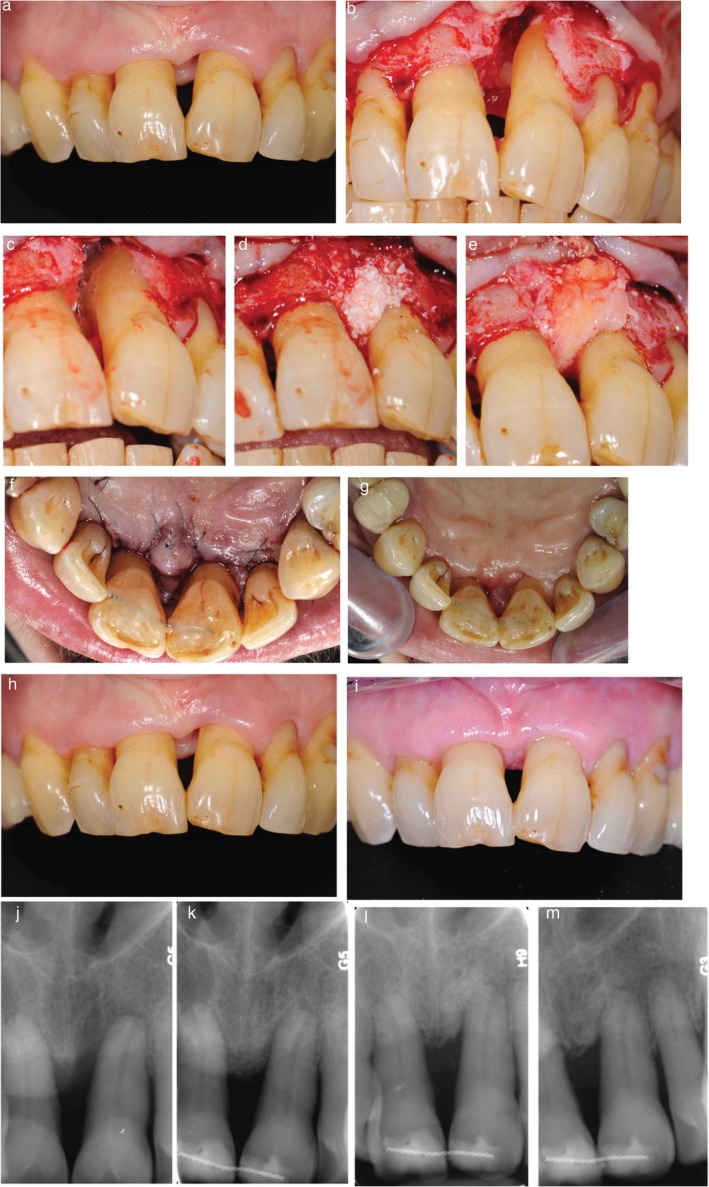
Case 3 (a) Preoperative view showing the presence of a gingival crater, resulting in a negative soft tissue architecture. (b–e) Infrabony defect extending beyond the apex (b); application of enamel matrix derivative and bone graft (c, d); placement of a contoured CTG as a “soft tissue roof” over the defect (e). (f) Immediate postoperative view showing primary closure. (g) Postoperative palatal view at 15 days, showing maintained interproximal closure. (h, i) Soft tissue changes from baseline to 36 months, showing conversion of the gingival crater into a stable, maintainable, and more favorable soft tissue architecture. (j–m) Radiographic follow‐up at baseline, 6 weeks, 9 months, and 36 months demonstrating substantial long‐term bone fill in the infrabony component.

- **Patient 1**: Narrow (≤ 3 mm wide), three‐wall infrabony defect on the mesial and palatal aspects of tooth 11. Baseline probing pocket depth (PPD) was 6 mm, and clinical attachment level (CAL) was 8 mm. Clinical features included an open gingival embrasure, a diastema, and buccal gingival recession (1–2 mm) affecting teeth 11 and 21. Gingival phenotype was thick, with ≥ 3 mm of keratinized tissue height (KTH) (Figure 1a).
- **Patient 2**: Narrow, two‐ to three‐wall infrabony defect on the mesial and palatal aspects of tooth 11. Baseline PPD was 8 mm, and CAL was 9 mm. Clinical examination revealed an extruded prosthetic crown on tooth 11, slight (≤ 1 mm) buccal gingival recession, and slight loss of interdental papilla height. The gingival phenotype was thick with ≥ 3 mm KTH (Figure 2a).
- **Patient 3**: Wide, predominantly one‐wall infrabony defect involving the buccal, mesial, and palatal aspects of tooth 21, extending to the apex. Baseline PPD was 8 mm and CAL was 13 mm. Notable clinical features included a large open gingival embrasure, a 2 mm‐deep interdental soft tissue crater, and generalized RT2–RT3 buccal gingival recession (3 mm) in the anterior maxillary sextant. Gingival phenotype was thick, with ≥ 3 mm KTH (Figure 3a). Although the defect extended to the apex radiographically, pulp testing confirmed vitality, and no endodontic treatment was indicated. Pulp status was monitored postoperatively. Teeth exhibiting Miller Class II or greater mobility were splinted immediately after surgery to enhance wound stability [17, 18].

All patients received comprehensive clinical examinations and necessary periodontal nonsurgical treatments prior to surgery. At the time of surgery, no residual probing depths ≥ 4 mm with bleeding on probing were present at sites other than those selected for treatment. Full‐mouth plaque scores were below 25%, and full‐mouth bleeding scores were below 10%.

### 2.2 Surgical Technique

All surgeries were performed by a single practitioner (MB). The surgical protocol, previously described by our group [12, 15], involves the combined use of enamel matrix derivative (EMD), deproteinized bovine bone mineral, and a CTG for the regenerative treatment of deep periodontal infrabony defects. In the present cases, a key modification was introduced: the CTG was contoured into T‐ or ice cream cone‐shaped configurations and strategically positioned as a “soft tissue roof” over the defect and a “soft tissue wall” along the buccal aspect. This design was intended to enhance soft tissue architecture in both the buccal and interproximal areas.

- Access and Defect Management: Defect access was achieved through a horizontal or oblique incision at the base of the papilla, following modified or simplified papilla preservation techniques [19, 20]. A coronally advanced flap (CAF) was designed using a split–full–split thickness approach, similar to that used for the treatment of multiple gingival recessions. The flap extended one to two teeth mesially and distally to ensure adequate elasticity [21].

A palatal beveled incision was performed to dissect the papilla overlying the defect, enhancing both contact surface and vascularization between the papilla and palatal flap. A deep horizontal incision was then made to separate the granulation tissue from the base of the papilla, facilitating interproximal soft tissue mobilization.

Flap elevation began on the buccal surfaces of the adjacent teeth. Subsequently, the papilla overlying the infrabony defect was gently elevated using tunneling instruments and shifted buccally to enhance access. Vertical releasing incisions were placed at the mesial and distal extensions of the flap, extending across the mucogingival junction to the alveolar mucosa. The angulation and symmetry of these incisions were carefully adjusted to create a trapezoidal flap design.

Granulation tissue was removed with curettes, and root surfaces were decontaminated using a combination of ultrasonic inserts and hand instruments. Root surface conditioning was performed using 24% EDTA gel for 2 min, followed by the application of enamel matrix derivative (EMD). The defect was then filled with deproteinized bovine bone mineral (Bio‐Oss, Geistlich Pharma, Switzerland).

- Connective Tissue Graft (CTG) Harvesting and Placement: A free gingival graft was harvested from the palatal mucosa and de‐epithelialized to obtain a CTG. The CTG was harvested directly in a T‐ or ice cream cone‐shaped configuration, then further trimmed and contoured for optimal adaptation to both the buccal and interproximal aspects of the defect (Figure 2c,d). The buccal component (“soft tissue wall”) was positioned adjacent to the defect and stabilized to the buccal papilla using internal mattress sutures with absorbable material. The interproximal portion (“soft tissue roof”) was extended into the defect and anchored to the palatal aspect using additional internal mattress sutures. Where needed, compressive sutures were secured to the periosteum to maintain precise graft adaptation and stability (Figure 2e). Finally, the flap was coronally repositioned to ensure complete, tension‐free coverage of the CTG, and the site was closed with internal mattress sutures for the papilla over the infrabony defect and sling sutures for the adjacent papillae.

### 2.3 Post‐Surgical Period and Periodontal Supportive Care

Post‐operative care included a 15‐day cessation period of brushing at the surgical site, the use of 0.2% chlorhexidine mouth rinses twice daily for 2–3 weeks, and the prescription of analgesics and amoxicillin at 2 g BID for 1 week [22]. Toothbrushing resumed following suture removal using an ultrasoft postoperative toothbrush (6.5/100e), with interdental brushes reintroduced between weeks 4 and 6 post‐surgery. All patients were enrolled in a periodontal supportive care program, with maintenance visits scheduled approximately two to three times per year during the follow‐up period.

### 2.4 Measurement and Analysis of Clinical Outcomes

The following clinical parameters were recorded using a UNC‐15 periodontal probe (UNC‐15, Hu‐Friedy, Chicago, IL, USA) at baseline and at multiple follow‐up visits:

- Probing pocket depth (PPD): Distance (mm) from the gingival margin to the base of the pocket at the deepest point of the interproximal site.
- Clinical attachment level (CAL): Distance (mm) from the cementoenamel junction (CEJ) to the base of the pocket at the same interproximal site.
- Buccal gingival recession (REC): Distance (mm) from the CEJ to the gingival margin on the buccal aspect.
- Papilla height (PH): Distance (mm) from the tip of the papilla to the most apical point of the CEJ of the involved tooth. If the CEJ was not detectable, the CEJ of the adjacent tooth was used as a reference [23].

Changes in each parameter from baseline to follow‐up were reported descriptively at the individual level. Due to the limited number of cases, no means, standard deviations, or statistical comparisons were calculated.

## 3 Results

At 15 days postoperatively, no patients reported prolonged bleeding or swelling, and no clinical complications such as infection or wound dehiscence were observed. Table 1 and Figures 1e, 2f summarize clinical outcomes over the 36 to 56 months follow‐up period. Representative clinical and radiographic images at baseline and post‐treatment are shown in Figures 1f–i, 2g–p, and 3f–k.

**TABLE 1:** Descriptive clinical parameters of the presented cases at baseline and at the last follow‐up.

Case	Gender, age	Follow‐up (m)	Tooth	PPD (mm)			CAL (mm)			REC (mm)			PI	
				T0	Last follow‐up	Reduction	Baseline	Last follow‐up	Gain	T0	Last follow‐up	Change	T0	Last follow‐up
1	F, 47y	55 m	11	6	2	−4	8	3	5	2	0	−2	Grade 1	Grade 2
2	F, 50y	56 m	11	8	2	−6	9	4	5	1	0	−2	Grade 3	Grade 3
3	F, 76y	36 m	21	7	3	−4	13	7	6	3	3	0	Gingival crater	Grade 0

Abbreviations: F, female; M, male; m, months; mm, milimeter; PI, Jemt papilla index; REC, buccal gingival recession; T0, baseline; y, year.

Patient 1: A 4 mm reduction in probing pocket depth (PPD), 5 mm CAL gain, and complete radiographic fill of the infrabony defect were achieved. From a mucogingival perspective, complete root coverage was obtained on the buccal aspect. Additionally, interdental papilla height increased by 2 mm, and spontaneous closure of the diastema resulted in near‐complete resolution of the initial black triangle.

Patient 2: PPD decreased by 4 mm, CAL improved by 5 mm, and complete radiographic bone fill was observed. Complete root coverage was achieved on the buccal aspect. Although a postoperative interproximal gingival recession resulted in an open embrasure at 15 days, papilla height improved between 6 and 24 months, partially resolving the black triangle. After 24 months, replacement of the prosthetic crown led to complete closure of the residual black triangle.

Patient 3: A 4 mm reduction in PPD, 6 mm CAL gain, and significant radiographic fill of the infrabony defect were obtained. The gingival margin remained stable on the buccal aspect, and notably, the initial interproximal gingival crater transformed into a positive soft tissue architecture that was maintained at 36 months.

According to the composite outcome measure (COM) proposed for evaluating periodontal regenerative therapy in infrabony defects [24], all treated sites achieved clinical success (COM1: CAL gain ≥ 3 mm and PPD ≤ 4 mm).

## 4 Discussion

In periodontics, two broad strategies are recognized for addressing the challenging management of the interdental papilla: papilla reconstruction, which aims to restore lost tissue volume and is typically performed in healthy, non‐inflammatory sites [25]; and papilla preservation, which seeks to maintain existing (intact or deficient) papilla architecture during surgical access, particularly in the context of PRT [8, 26, 27].

The present case report introduces a c‐CTG approach that integrates infrabony defect regeneration with targeted soft tissue optimization. In this technique, the CTG is strategically contoured and positioned as a “soft tissue roof” over the defect to achieve both regenerative success and improved buccal and interdental soft tissue profiles. This approach aligns with the growing trend of using contoured CTGs or biomaterials to augment interproximal soft tissues around teeth and dental implants [28, 29, 30, 31, 32, 33].

Achieving and maintaining primary wound closure is critical to protect the surgical site, stabilize the blood clot, prevent postoperative complications, and allow periodontal regeneration to occur [34]. Flap tension plays a decisive role—excessive closing forces significantly increase the risk of wound dehiscence [35]. To address these challenges, several strategies were implemented. First, the flap design was adapted from the frontal variation of the coronally advanced flap (CAF) envelope described by Zucchelli and De Sanctis, originally developed for the management of bilateral multiple adjacent gingival recessions involving the central and lateral incisors [36]. In this modification, the flap's axis of rotation passes through the interincisal papilla, allowing maximum flap advancement over the interdental area corresponding to the infrabony defect. Second, meticulous care was taken to achieve complete flap release prior to closure using periosteal releasing incisions and vertical incisions when necessary. Park et al. demonstrated that periosteal releasing incisions combined with two vertical incisions can achieve a mean flap extension of 13.8 ± 3.2 mm, corresponding to more than one and a half times the flap's original length [37]. We believe this extension provided sufficient mobility to cover the connective tissue graft (CTG) in the interdental region without tension. Third, fine sutures (7–0) were used for the interdental papilla covering the defect. As demonstrated by Burkhardt et al., tissue trauma induced by suture tension can be minimized by using smaller‐diameter sutures, thereby supporting better wound stability and healing [38]. We believe that the combination of these strategies was fundamental in maintaining tension‐free primary closure throughout the healing phase (Figures 1e,f, 2g,h, 3g). Importantly, wide and thick interdental papillae offer greater connective tissue volume and vascular supply [39]. Therefore, this technique is best indicated for interdental distances of ≥ 2 mm, where improved access and perfusion can be ensured.

Across all cases, this combined technique resulted in favorable regenerative outcomes, with CAL gain and radiographic bone fill maintained throughout follow‐up intervals of up to 56 months. In addition, improvements in papillary height and contour were observed, suggesting that papilla reconstruction may be achieved concurrently with regenerative therapy of infrabony defects, rather than through secondary, staged soft tissue surgeries. We deliberately avoided a staged approach to minimize patient morbidity associated with additional surgery and because the use of CTG as a separate, delayed procedure does not guarantee more predictable papilla reconstruction [25].

The success of this approach may be attributed to the mechanical support provided by the c‐CTG, which acts as a volumizing scaffold at the base of the papilla. This mechanical effect likely facilitates favorable tissue maturation and may contribute to long‐term improvement through a creeping attachment phenomenon [40]. This hypothesis is supported by evidence correlating increased gingival thickness with greater papilla height [41]. Similar interproximal CTG augmentation procedures have recently been described in the literature. A comparable outcome was reported in a 12‐month follow‐up case, where a CTG was positioned buccally at the base of the papilla [42]. However, in that report, the CTG base technique was performed as a secondary procedure following a failed regenerative treatment.

Notably, the complete closure of black triangles observed in Cases 1 and 2 was not solely attributable to soft tissue changes. In Case 1, spontaneous closure of a diastema occurred following surgical therapy, contributing to papillary fill. In Case 2, replacement of the prosthetic crown, with apical repositioning of the contact point, was essential for complete closure [43]. These findings emphasize the multifactorial etiology of papilla loss and reinforce the importance of integrating restorative and orthodontic considerations when planning for esthetic outcomes [44, 45].

Spontaneous tooth repositioning after periodontal treatment, as seen in Case 1, is a well‐documented phenomenon. Gaumet et al. reported that 69.7% of sites demonstrated diastema reduction after therapy, with complete closure observed in 51.5% [46]. Another study has shown that surgical therapy may enhance repositioning, particularly in cases of minor (< 1 mm) pathologic migration [47], likely due to resolution of inflammation, wound contraction, and remodeling of transeptal fibers [48, 49, 50, 51].

In Case 2, despite CTG placement, early interproximal recession and persistent black triangle formation were observed. This outcome was likely due to unfavorable anatomical factors, including crown overeruption and tooth malposition—both of which are known contributors to papilla loss [25]. Progressive improvement of soft tissue contour was observed over time, supporting the notion that tissue remodeling via creeping attachment is a slow process that may extend beyond 12 months [40, 52].

Case 3 presented the most challenging scenario, with a non‐contained infrabony defect extending to the apex. PRT resulted in substantial CAL gain and tooth retention over 30 months, supporting evidence that regenerative treatment can change the long‐term prognosis of teeth previously considered “hopeless” [53]. Although the esthetic improvement was limited due to a residual open embrasure, conversion of a deep gingival crater into a flat, maintainable soft tissue contour may offer biological benefits by facilitating local plaque control and reducing inflammation risk [54].

Of particular note, pulp vitality was preserved in this case, throughout the follow‐up period despite the proximity of the defect to the apex. This aligns with emerging evidence challenging the need for routine preventive endodontic treatment in such cases [55, 56]. When clinically feasible, preserving pulp vitality may reduce treatment cost and complexity, while also contributing to greater tooth longevity by maintaining structural integrity and adaptability to occlusal load [57, 58].

This case report is inherently limited by its descriptive nature and the small number of cases. While the results suggest feasibility and potential benefits of the proposed procedure, larger controlled studies are needed to assess its reproducibility, long‐term stability, and key predictors of success. Future refinements may include coronal suspension sutures or interdental splints to further elevate and support the papilla during healing [42]. However, these modifications must be carefully balanced against the risk of increased flap tension, which may predispose to dehiscence and compromise wound healing.

## 5 Conclusions

Within the limitations of this case report, the combination of infrabony defect reconstruction with connective tissue grafting as a “soft tissue roof” appears to be an effective strategy for achieving both favorable regenerative outcomes and improvements in buccal and interdental soft tissue contours. In two of the three presented cases, restorative interventions (diastema closure, crown replacement) likely influenced interproximal soft tissue outcomes, highlighting the critical importance of a multidisciplinary approach for the esthetic management of deficient papillae. This technique might be beneficial for managing infrabony defects associated with buccal and interdental gingival recession. However, these encouraging findings require validation through controlled studies with larger sample sizes.

## Funding

The authors have nothing to report.

## Consent

Before initiating the treatment, the expected outcomes were explained, and oral informed consent was obtained from the patients.

## Conflicts of Interest

The authors declare no conflicts of interest.

## Data Availability

The data that support the findings of this study are available on request from the corresponding author. The data are not publicly available due to privacy or ethical restrictions.
